# The Neuroscience of Organizational Trust and Business Performance: Findings From United States Working Adults and an Intervention at an Online Retailer

**DOI:** 10.3389/fpsyg.2020.579459

**Published:** 2021-01-11

**Authors:** Rebecca Johannsen, Paul J. Zak

**Affiliations:** Center for Neuroeconomics Studies, Claremont Graduate University, Claremont, CA, United States

**Keywords:** job satisfaction, employee well-being, discretionary effort, neuromanagement, organizational culture

## Abstract

This paper reports findings from a nationally representative sample of working adults to quantify how a culture trust improves business performance. Analysis of the national sample showed that organizational trust and alignment with the company’s purpose are associated with higher employee incomes, longer job tenure, greater job satisfaction, less chronic stress, improved satisfaction with life, and higher productivity. Employees working the highest quartile of organizational trust had average incomes 10.3% higher those working in the middle quartile of trust (*p* = 0.000) indicating that trust increases productivity. In order to demonstrate the causal effect of trust on business performance, we created an intervention to increase organizational trust in a division facing high job turnover at a large online retailer. The intervention increased organizational trust by 6% and this improved job retention by 1%. These studies show that management practices that increase organizational trust have salubrious effects on business performance.

## Introduction

Peter Drucker challenged traditional labor economics by arguing that “knowledge workers” (Drucker, 1988, p. 3) have significant power when choosing for whom they work, where they work, and what they work on (Drucker, 1988). At the time of Drucker’s writing, and even today, many companies de-motivate their “human capital” by treating them like capital rather than humans. Traditional labor economics presumes that work provides disutility and therefore employees are expected to shirk whenever possible (Spencer, 2015). Companies that design management processes assuming there is this conflict between employees and supervisors often create such conflicts.

The traditional labor economics view runs counter to an accumulation of evidence showing that human-centric organizations have higher productivity and lower job turnover rates (Rastogi, 1986; Vandenberghe, 1999; Hall and Yip, 2016; Jena et al., 2018). Companies have discovered that employees often quit to take jobs that are more creative, exciting, and energizing (Bersin, 2014). Turnover is a particularly expensive problem as most jobs require firm-specific skills that cannot be transferred and can take years to cultivate (Bersin, 2014). Unlike machinery, people can exert discretionary effort if motivated to do so. Beyond monetary compensation, many colleagues desire autonomy, honesty, appreciation, and work that has a positive impact on their communities (Zak, 2017).

These conditions are part of a company’s “culture” defined as a set of employee behaviors that occur at work (Deloitte., 2014). Often, companies write mission statements, bring in designers to create interesting work spaces, and create recognition programs in order to establish an organizational culture without having clear guidance on which aspects of culture create value; value both from the employee’s perspective and for the organization as a whole. Culture is often ignored because it seems ambiguous or difficult to manage. We propose herein that a set of behaviors that create trust between work colleagues is an aspect of organizational culture that improves business outcomes including productivity, job satisfaction, turnover, and well-being. We also show that organizational trust can measured and managed like other business process.

## Theory

### Organizational Trust and Oxytocin

The motivation for our focus on organizational trust is research showing that countries with high generalized trust have faster income growth than low trust countries. Trust reduces transaction costs and thereby facilitates wealth creation (Zak and Knack, 2001; Slemrod and Katuscak, 2005; Pucetaite and Lamsa, 2008; Dincer and Uslaner, 2010; Bjornskov, 2012; Kong, 2013). Interpersonal trust also contributes to individual well-being by facilitating secure attachments to others (Zak, 2017; Zak and Fakhar, 2006; Slemrod and Katuscak, 2005). Yet, little is known about the effects of organizational trust on business performance. On a national level, generalized trust is high when formal and informal institutions function efficiently and fairly, when income distribution is relatively equal, and when incomes are high (Zak and Knack, 2001). These factors can be changed through policies, often producing a positive return on the cost of policy changes by generating faster income growth (Knack and Zak, 2003; Slemrod and Katuscak, 2005). Similar to citizens in a country who interact and trade with each other, workplace colleagues interact with each other repeatedly, and the quality of these interactions is affected by the organization’s culture; that is, its formal and informal institutions. The types of colleague interactions may build or degrade trust.

When seeking to understand how trust varies in organizations, we drew on research showing that the neurochemical oxytocin (OT) is released in the brain after positive interactions with others, including strangers, and signals that the other person appears to be trustworthy (Zak et al., 2004, 2005, 2008; Morhenn et al., 2008; Barraza and Zak, 2013; Zak and Barraza, 2013; reviewed in Zak, 2012). Infusing synthetic OT into human brains substantially increases trust in a sequential dyadic money transfer task (Kosfeld et al., 2005), increases generosity and charity (Zak et al., 2007; Barraza et al., 2011), and can help rebuild trust after a breach (Baumgartner et al., 2008). The neuroscience research shows that OT binding to neurons in the subgenual cortex stimulates the release of midbrain dopamine (Love, 2014). This means that being trustworthy makes people feel good and when this happens at work, work itself may be enjoyable. The types of prosocial behaviors that induce OT release are found in employees who are good organizational citizens (Battistelli et al., 2013; Steger et al., 2012). Organizations that create work environments that stimulate OT production among colleagues are expected to have high-trust cultures.

Our group ran laboratory experiments to assess how positive and negative citizenship behaviors in simulated work settings affected neurophysiology, motivation, and productivity (Alexander et al., 2018; Terris et al., 2018; Kraig et al., 2019; Johannsen and Zak, 2020). We then gained permission from a set of businesses and nonprofit organizations to measure employees’ neurophysiology, motivation, and productivity in their workplaces (Zak, 2017). We combined these findings with a review of the literature indicating ways that social interactions stimulate the brain to produce OT. We used all this information to identify eight behaviors that organizations can influence that may affect organizational trust (”Trust” herein). To make these easier to remember, we created an acronym OXYTOCIN to represent them; this stands for Ovation, eXpectation, Yield, Transfer, Openness, Caring, Invest, and Natural. A definition of each and brief rationale for inclusion are presented next, while a full explanation and justification can be found elsewhere (Zak, 2017).

The first factor, Ovation celebrates the contributions of high performers. When Ovation is close in time to when a goal is met, is public, comes from peers, is tangible and unexpected, then the likelihood and magnitude of OT release are higher than when recognition does not have these aspects (Zak, 2018). The second component, eXpectation gives colleagues concrete, difficult but achievable goals. Such goals typically require that colleagues draw on the social resources at work, increasing the chances for OT release (Barraza and Zak, 2009; Zak, 2018). Yield empowers colleagues to execute projects as they see fit, increasing ownership over outcomes (Argyris, 1976; Stajkovic and Luthans, 1998; Kirkcaldy et al., 2002; Campbell et al., 2011). This demonstrates trust by a supervisor who must provide consistent feedback so projects stay on track. When feedback is positive, OT release is likely. Transfer allows colleagues to job-craft by choosing the tasks, projects, work location, and hours they prefer (Deci and Ryan, 2000; Katz and Krueger, 2017). This signals trust while holding colleagues accountable to reach goals. Openness about the organization’s goals and the reasons for management decisions reduces the stress colleagues absorb on the job and thereby increases the brain’s ability to produce OT (Pucetaite and Lamsa, 2008; Zak, 2018). Caring creates opportunities for colleagues to intentionally build relationships with each other, stimulating OT release and enhancing teamwork (Alexander and Zak, 2015). A culture of Invest expends resources to stimulate colleague professional and personal growth (Ryff and Keyes, 1995). This shows the organization expects the employee to remain on the job at least in the medium term since it is providing opportunities for growth. Lastly, Natural is trustworthy behavior by leaders. When trust is modeled by those in charge, others tend to follow, and reciprocal OT release is likely to occur (Zak et al., 2004, 2005; Sendjaya and Pekerti, 2010).

We tested the validity of these factors by returning to workplaces and inviting employees take the survey we had developed. This provided preliminary evidence that the OXYTOCIN factors captured behaviors that contribute to organizational trust.

### Purpose

There are at least two types of purpose in organizations. The first is transactional purpose, the processes built to execute the generation of revenue and to pay expenses (Daft, 2016). Here we will focus on a second type that follows from work by Peter Drucker, W. Edwards Deming and others who argue that the purpose of an organization is to improve the lives of its employees and customers (Drucker, 1993/1946, 2007/1955; Deming, 1994; Steger et al., 2012; Deloitte., 2014). We will call this aspect of an organization its transcendent purpose (denoted “Purpose”). Purpose is necessarily other-focused, invoking the inherent service to others that is at the core of all organizations. When the Purpose of an organization or task is known, then effort and productivity are higher (Chandler and Kapelner, 2013; Brown et al., 2015). While Trust captures the dynamics of team interactions, Purpose tells the team why it is going forward. Without the who and why in place, team performance suffers (Kirkman and Rosen, 1999; Zak, 2018; Fahn and Hakenes, 2019). Organizations with understood and lived Purposes have higher retention and are more profitable (Harter et al., 2002; Deloitte., 2014). Research from our group has shown that tasks with Purpose reduce physiologic arousal, consistent with the actions of OT (Kraig et al., 2018). We therefore hypothesized that, along with Trust, an understanding of the organization’s Purpose is another physiologic route through which colleagues induce OT release in each other.

### Causal Model

**FIGURE 1:**
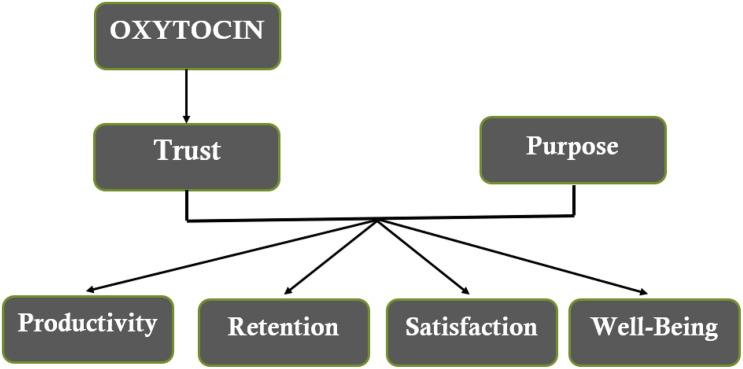
A causal model relating Trust and Purpose to individual and organizational outcomes.

Figure 1 presents a schematic model that identifies the causal relationships we will test, associating Trust and Purpose with business outcomes. We will test this model in two ways. First, by measuring the cross-section variation in Trust and Purpose and associating these with performance measures. Second, we will analyze the effect of actively changing Trust to establish causation.

## Study 1: National Data

The first test of the model uses a nationally representative sample of United States working adults collected in February 2016 by the survey company Qualtrics. It matches United States demographics for age, sex, ethnicity, and geographic dispersion, with 1,095 respondents from all 50 states. The majority of the sample, 79%, consists of people working in the for-profit sector with the remainder working for government and non-profits. The survey was designed to test if a culture of Trust and Purpose affects individual and company performance. This paper will use multiple dependent variables to generate convergent evidence that these aspects of culture drive performance. The Institutional Review Board of Claremont Graduate University approved this study and all participants provided informed consent before inclusion.

### Variables

The national survey collected information about demographics, behaviors and attitudes toward work, and quality of life. To quantify organizational trust, the eight OXYTOCIN factors were assessed and averaged. Purpose is measured as the average of three questions about personal alignment with the organization’s values (see Appendix).

The dependent variables are productivity, retention, joy at work (“Joy”), job satisfaction, closeness to colleagues, satisfaction with life (SWL), chronic stress, and depersonalization. Productivity is measured in two ways. The first is by self-report, although these data are suspect as the median response is 95 out of 100. To overcome this bias, the second way uses a subset of responses to the Utrecht Work Engagement Scale called “vigor” as a proxy for productivity following previous research (Schaufeli and Bakker, 2004; Schaufeli et al., 2006; De Bruin et al., 2013; White et al., 2014). Vigor assesses how much energy colleagues have during work tasks, how immersed people are in their work, if they feel energized while working, if they can work for long periods of time, and if they are mentally resilient at work. Retention is determined from responses about the likelihood of continuing to work for the organization for the next 12 months. Job satisfaction is ranked on a 7-point scale, closeness to colleagues is measured using the Inclusion of Others in Self survey (IOS; Aron et al., 1992), and well-being was captured using the SWL survey (Diener et al., 1985). Chronic stress was measured on a seven-point scale, while depersonalization is part of the Maslach Burnout Inventory (MBI; Maslach et al., 1996). Depersonalization has been shown to be a significant indicator of chronic stress and burnout (Maslach and Jackson, 1981; Leiter and Maslach, 1999). Joy is measured by averaging two questions from Zak (2017) that ask how much one enjoys working on a typical day. Income is measured in $25,000 intervals.

Control variables include the number of hours worked per week, age, and marital status. We also assessed participants’ conscientiousness as a personality trait using the Big Five Inventory (BFI; John and Srivastava, 1999). This trait has been associated with higher levels of productivity, job-satisfaction, and well-being (Dobewall et al., 2013; Berglund et al., 2015; Cubel et al., 2016).

### Analytical Approach

Data analysis was performed using t-tests of differences in means, correlations, and least squares regressions. We also estimated ordered-logistic and log-log regressions to determine predictive accuracy and size effects (elasticities). The data were analyzed in aggregate, as well as broken into categories, such as organization type, and high-trust and low-trust groups in order to present convergent evidence for our hypotheses.

## Results: National Data

Descriptive statistics are presented in Table 1 for the OXYTOCIN components, dependent, independent, and control variables.

**TABLE 1:** Descriptive statistics for the national sample.

**Type**	**Variable**	**Mean**	**SD**	**Min**	**Max**
Independent	Ovation	3.815	1.252	1	6
Independent	eXpectation	4.278	1.405	1	6
Independent	Yield	3.996	1.173	1	6
Independent	Transfer	4.315	1.379	1	6
Independent	Openness	3.941	1.245	1	6
Independent	Caring	4.133	1.417	1	6
Independent	Invest	4.161	1.377	1	6
Independent	Natural	4.143	1.424	1	6
Independent	Organizational Trust	4.216128	1.135205	1	6
Independent	Purpose	4.3188	1.32118	1	6
Dependent	Productivity (Vigor)	5.170002	1.356382	1	7
Dependent	Productivity	77.32694	24.24813	0	100
Dependent	Retention	4.84275	1.448044	1	6
Dependent	Job Satisfaction	4.59726	1.408836	1	7
Dependent	Closeness to Colleagues	3.234703	1.507047	1	6
Dependent	Joy	4.575576	1.2258	1	6
Dependent	Well-being	15.2188	6.64217	1	28
Dependent	Chronic Stress	2.677479	1.18226	1	6
Dependent	Income	3.374 ($59,249)	1.78564	1 (<$25,000)	8 ($200,000+)
Control	Sex	0.5022831	0.5002233	0	1
Control	Hours worked/week	2.36803 (43.68)	0.84475	1 (<20)	5 (>60)
Control	Age	3.08432 (35–44 years)	1.514434	1 (18–24)	6 (65+)
Control	Married	0.456621	0.4983423	0	1
Control	Personality (conscientiousness)	4.088245	0.6193216	1	5

### OXYTOCIN and Trust

All eight OXYTOCIN factors were highly correlated with trust: (Ovation *r* = 0.735; eXpectation *r* = 0.906; Yield *r* = 0.630; Transfer *r* = 0.892; Openness *r* = 0.734; Caring *r* = 0.890; Invest *r* = 0.840; Natural *r* = 0.880; *t*-tests, *p*s < 0.0000). Each factor is given equal weight when measuring organizational trust. In this sample, Ovation had the lowest average score and Transfer had the highest. Assessing reliability of the measures using Cronbach’s alpha, we find α = 0.93.

### Productivity and Retention

Productivity and retention directly increase profit and shareholder value and are therefore the first outcomes we analyzed. Trust increased productivity measured both by vigor from the Utrecht engagement scale (*r* = 0.55, *p* = 0.000) and by self-report (*r* = 0.51, *p* = 0.000). Purpose also positively increased both productivity measures (vigor: *r* = 0.60, *p* = 0.000; self-report: *r* = 0.50, *p* = 0.000). Similarly, higher levels of Trust and Purpose increased colleague retention (Trust: *r* = 0.57, *p* = 0.000; Purpose: *r* = 0.56, *p* = 0.000). The partial correlations from Trust and Purpose on these outcome measures are similar when hours worked, personality, age, income, sex, and marital status are included as controls (Table 2 and Appendix).

**TABLE 2:** Trust continues to be positively related productivity and retention when control variables are included.

**Variables**	**(1)**	**(2)**	**(3)**
	
	**Productivity (Vigor)**	**Productivity (Self-Report)**	**Retention**
Organizational Trust	0.168**	4.036**	0.431**
	(0.0380)	(0.748)	(0.0466)
Purpose at Work	0.335**	3.907**	0.330**
	(0.0321)	(0.632)	(0.0394)
Weekly Hours	0.126**	−0.685	0.0822
	(0.0370)	(0.729)	(0.0445)
Personality	0.676**	10.71**	0.0772
	(0.0551)	(1.084)	(0.0679)
Age	0.0918**	1.427**	0.0778**
	(0.0208)	(0.408)	(0.0250)
Income	0.000358	−0.600	0.0132
	(0.0188)	(0.369)	(0.0228)
Female	−0.122*	0.0398	0.0999
	(0.0614)	(1.207)	(0.0739)
Married	−0.101	1.223	0.0206
	(0.0650)	(1.278)	(0.0784)
Constant	−0.196	−1.083	0.722**
	(0.214)	(4.210)	(0.263)
Observations	1,078	1,078	1,054
R-squared	0.486	0.358	0.384
VIF	1.47	1.47	1.47

*Standard errors in parentheses. ^∗∗^*p* < 0.01, ^∗^*p* < 0.05.*

The size effects of an increase in Trust or Purpose are moderately high. An organization that increased Trust by 10% would see a rise in productivity of 1.57% for vigor and by 4.50% using the self-report data. Similarly, a 10% increase in Purpose would raise productivity between 2.38% (vigor) and 2.72% (self-report). Analyzing the impact of an increase in Trust and Purpose on retention shows that a 10% increase in either would cause a 3.9% increase in retention.

### Income

Trust had a positive effect on colleague earnings (*r* = 0.10, *p* = 0.0011). Those in the highest Trust group reported average incomes of 10.3% higher than the middle group (*p* = 0.000) and 11.63% higher than lowest trust group (*p* = 0.000). In competitive labor markets, productivity is closely related to earnings. The higher earnings by colleagues in high Trust organizations indicates that they are more productive than those working on lower Trust companies.

### Job Satisfaction, Joy, and Colleague Closeness

Consistent with our hypotheses, Trust increased job satisfaction (*r* = 0.59, *p* = 0.000) and closeness to colleagues (*r* = 0.40, *p* = 0.000). Purpose also had a positive impact on both job satisfaction (*r* = 0.57, *p* = 0.000) and closeness to colleagues (*r* = 0.36, *p* = 0.000). The quantitative effect of raising Trust and Purpose are similar when hours worked, personality, age, income, sex, and marital status are included as controls (Table 3 and Appendix). A 10% increase in Trust is associated with a 4.5% increase in job satisfaction and 4% greater closeness to colleagues. The impact of an increase in Purpose is similar: a 10% increase would result in 2.6% more job satisfaction and 1.7% greater closeness to colleagues.

**TABLE 3:** Trust and Purpose have a positive relationship on job satisfaction and colleague closeness when controls are included.

**Variables**	**(4)**	**(5)**
	
	**Job Satisfaction**	**Closeness with Colleagues**
Organizational Trust	0.439**	0.348**
	(0.0431)	(0.0545)
Purpose at Work	0.295**	0.135**
	(0.0364)	(0.0461)
Weekly Hours	0.0391	−0.0512
	(0.0420)	(0.0531)
Personality	0.0714	0.246**
	(0.0624)	(0.0791)
Age	0.0494*	−0.0417
	(0.0235)	(0.0298)
Income	−0.0564	0.169
	(0.0736)	(0.0932)
Female	0.0417	−0.278**
	(0.0695)	(0.0880)
Married	0.0893**	0.0331
	(0.0212)	(0.0269)
Constant	0.654**	0.386
	(0.242)	(0.307)
Observations	1,078	1,078
R-squared	0.406	0.188
VIF	1.47	1.47

*Standard errors in parentheses. ^∗∗^*p* < 0.01, ^∗^*p* < 0.05.*

The neuroscience predicts a nonobvious aspect of high-Trust and high-Purpose cultures: people will enjoy working at them. Both Trust and Purpose are highly correlated with Joy (Trust: *r* = 0.78, *p* = 0.000; Purpose: *r* = 0.75, *p* = 0.000). The science also predicts that Trust and Purpose reinforce each other at work and the data support this with a high correlation between Trust × Purpose and Joy (*r* = 0.80, *p* = 0.000). If a company were able to increase Trust by 10%, this would raise Joy by 5% (*p* = 0.000). Similarly, a 10% increase in Purpose would positively impact Joy by 3.1% (*p* = 0.000). If organizations instituted programs to increase both Trust and Purpose by 10%, Joy would rise by 7.8% (*p* = 0.000).

### Chronic Stress and Satisfaction With Life

Chronic stress and SWL are primary indicators linked to well-being (Binder and Ward, 2013; Berglund et al., 2015; Kjell et al., 2016). Chronic stress drives job turnover and inhibits one’s SWL outside of work. We tested the effect of Trust and Purpose on job burnout and depersonalization using the Maslach Burnout Inventory. We found that Trust reduced chronic stress (*r* = -0.42, *p* = 0.000) and depersonalization (*r* = -0.37, *p* = 0.000) and Purpose had similar effects (stress: *r* = -0.32, *p* = 0.000; depersonalization: *r* = -0.35, *p* = 0.000). As expected, both factors had positive effects on SWL (Trust: *r* = 0.36, *p* = 0.000; Purpose: *r* = 0.39, *p* = 0.000). These results continue to be significant and are of similar magnitude when control variables are included (Table 4 and Appendix).

**TABLE 4:** Trust and Purpose continue to be positively associated with well-being and Joy and are mostly negatively associated with stress and depersonalization when controls are included.

**Variables**	**(6)**	**(7)**	**(8)**	**(9)**
	
	**Well-Being**	**Joy**	**Chronic Stress**	**Depersonalization**
Organizational Trust	0.636**	0.509**	−0.380**	−1.630**
	(0.224)	(0.0275)	(0.0422)	(0.349)
Purpose at Work	0.898**	0.331**	−0.00547	−0.910**
	(0.190)	(0.0232)	(0.0353)	(0.295)
Weekly Hours	0.0624	0.0470	0.200**	1.108**
	(0.219)	(0.0266)	(0.0404)	(0.340)
Personality	2.176**	0.180**	−0.233**	−2.648**
	(0.325)	(0.0398)	(0.0604)	(0.505)
Age	−0.379**	0.0336*	−0.120**	−1.286**
	(0.122)	(0.0149)	(0.0227)	(0.190)
Income	0.628**	−0.0122	0.0169	−0.0440
	(0.111)	(0.0135)	(0.0205)	(0.172)
Female	0.110	0.110*	−0.111	−0.587
	(0.362)	(0.0440)	(0.0671)	(0.563)
Married	2.530**	0.0289	−0.0331	−0.355
	(0.383)	(0.0466	(0.0711)	(0.596)
Constant	−2.500*	0.0279	5.172**	41.47**
	(1.263)	(0.154)	(0.235)	(1.963)
Observations	1,078	1,073	1,065	1,078
R-squared	0.286	0.687	0.240	0.222
VIF	1.47	1.48	1.47	1.47

*Robust standard errors in parentheses. ^∗∗^*p* < 0.01, ^∗^*p* < 0.05.*

Assessing elasticities, an increase in Trust by 10% would reduce chronic stress by 4.7%, decrease depersonalization by 3.3%, and increases SWL by 1.5%. A 10% increase in Purpose would have similar effects, reducing chronic stress by 0.10%, diminishing depersonalization by 1.7%, and increasing SWL by 2.4%.

### Non-profits

People working for non-profit organizations had the same Trust but higher Purpose compared to colleagues in for-profit businesses. Purpose was 10.2% higher (*p* = 0.001) compared to private industry. This produced greater job satisfaction for those working in nonprofits, 6.2% higher than employees in businesses (*p* = 0.039). At the same time, organizations in the social sector have 3.56% (*p* = 0.066) lower productivity than for-profit businesses and consequentially the average nonprofit employee earns 8.6% less (p = 0.089) than employees in businesses.

## Discussion: National Data

Our findings have demonstrated that organizations with cultures that empower colleagues with Trust and Purpose perform significantly better than their peers. This improved performance is due to greater colleague productivity, lower turnover, and higher satisfaction at work and at home. Traditional economic theory pits the interests of organizations and employees against each other, where firms must continually tweak incentive schemes and constantly monitor employees to ensure effort at work (Fehr and Gachter, 2000; James et al., 2011; Spencer, 2015). Counter to this perspective is the labor market gift exchange model developed by Akerlof (1982) in which work colleagues respond to the gift of wages offered by the firm by providing discretionary effort to their employers (Mahmood and Zaman, 2010; Akerlof, 1982; Fehr and Gachter, 2000). Our analysis shows that Trust may also be viewed by work colleagues as a gift, generating higher productivity and longer job tenure. This is corroborated by our finding that high-trust organizations reciprocate additional effort by paying colleagues a premium over wages at lower-trust companies. Organizations in the highest quintile of Trust pay employees 10.3% more than employees working in companies in the middle quintile of Trust and 11.6% more than the quintile of the lowest Trust companies (*p*s < 0.05). Demographics were not significantly different between the Trust quintiles, with age, sex, and race being similarly distributed in each (Appendix). The United States labor market is highly competitive so higher wages indicate that high-trust companies are more profitable than low-trust ones. This is confirmed in findings for productivity. Colleagues working in the highest Trust organizations report productivity that is more than 250% (*p* = 0.000) higher than the lowest Trust quintile and 50% (*p* = 0.000) more than the middle quintile.

The productivity difference in high-trust organizations indicates that colleagues are exerting discretionary effort. Joy at work often comes when people make progress on projects (Amabile and Kramer, 2011) and the greater productivity of high-Trust organizations also produces Joy. Colleagues working in companies in the highest quartile of Trust had 21.7% more Joy than the middle quartile and 89.7% more Joy than the lowest Trust quartile. These effects suggest that company culture promotes effective teamwork.

Job satisfaction is another measure of the intrinsic and extrinsic value people receive from work (Mirvis and Hackett, 1983; White et al., 2014). Satisfaction at work has positive effects on both productivity (*r* = 0.55, *p* = 0.000) and turnover (*r* = -0.44, *p* = 0.000) as others have shown (Ertürk and Vurgun, 2015; Frederiksen, 2016), creating value for organizations. Job satisfaction for respondents working in the top Trust quintile was 42% (*p* = 0.000) higher than those working in bottom group and 17% (*p* = 0.000) higher compared to those in the middle quintile. Our analysis also showed that non-profit colleagues have 6.2% (*p* = 0.001) higher job satisfaction than those working in private sector. This confirms other research showing that working for a nonprofit organization produces higher job satisfaction compared to employees in for-profit companies (Mirvis and Hackett, 1983). We traced this effect to a greater understanding of the organization’s Purpose than was found among for-profit employees. More generally, job satisfaction can be increased in for-profits and non-profits by raising Trust or Purpose. For example, those working in the highest Trust private sector quartile had 16.4% (*p* = 0.000) more job satisfaction than the average for non-profit colleagues.

Employee retention is critical for firm performance due to the high costs associated with recruiting, on-boarding, and training new employees, as well as the value of firm-specific knowledge that accumulates on the job (Meister, 2012; Bersin, 2014; Mason and Bishop, 2015). Recruiting and on-boarding averages nearly one year annual salary for professional positions (Tracey and Hinkin, 2008; Merhar, 2016). The analytics herein show that Trust has a significant impact on the intention of colleagues to remain with their current employer. Fully 95% of respondents in the highest Trust quintile planned to stay with their employer for the next year (*p* = 0.000). This significantly reduces costs for these employers. Conversely, in the lowest Trust quintile, 51% (*p* = 0.000) planned to leave employment in the next year. Accordingly, respondents in the highest Trust quintile have 28 months longer job tenure than the lowest quintile (*p* = 0.000). In addition to more productive colleagues at high-Trust organizations, greater retention also gives them a competitive edge.

We also found that organizational trust had a positive effect on employees’ lives outside of work. Those working in highest Trust quintile had 16.2% (*p* = 0.000) greater life satisfaction than the middle quintile and 54.5% (*p* = 0.000) more life satisfaction than the lowest group. One way Trust improves life satisfaction is by reducing chronic stress. Colleagues who moved from the lowest Trust quintile to the middle quintile would face 15.8% less chronic stress (*p* = 0.000); moving from the middle to the highest Trust quintile would reduce chronic stress by 34.5% (*p* = 0.000). Not only did Trust reduce chronic stress, it was associated with overall improvements in health. Our analysis shows that colleagues with jobs in the highest Trust quintile took 8.5 (*p* = 0.047) fewer sick days compared to the middle Trust quintile. Overall health for those in the highest quintile of Trust was 13% better than those in the middle quintile (*p* = 0.000) and 17% better overall than the lowest group (*p* = 0.000). These findings taken as a whole suggest that Trust helps to align the incentives of organizations and employees.

Our analysis showed that Purpose also affects the performance of organizations. This starts with a clear Purpose statement and processes that keep Purpose top of mind for colleagues. This can be done by stating the organization’s Purpose at the beginning of meetings, and displaying it on posters, screensavers, or apps. For example, the management consulting firm KPMG built an app called “10,000 stories” so colleagues could share the positive ways they have improved their clients’ lives (Pfau, 2015). They expected it could take years to collect 10,000 stories; they reached this goal in 2 months. Within a year, 42,000 stories had been collected. The annual KPMG partner survey completed after the 10,000 stories app launch showed that 90% of respondents reported this Purpose initiative increased their pride in working for KPMG.

Purpose directly improves job satisfaction, productivity, Joy, retention and well-being (*r* = 0.57, *p* = 0.000; *r* = 0.57 *p* = 0.000; *r* = 0.75, *p* = 0.000; *r* = 0.56, *p* = 0.000; *r* = 0.37, *p* = 0.000). For example, those working in highest quintile of Purpose organizations are 66.4% more satisfied with work than the lowest quintile (*p* = 0.000) and 22.6% more satisfied than the middle quintile (*p* = 0.000). Similarly, people working in the highest Purpose organizations experience 20.2% more Joy than those in the middle quintile (*p* = 0.000) and 81.6% more Joy than employees in the lowest quintile (*p* = 0.000). High Purpose also increases outside-of-work life satisfaction. SWL is 15.2% higher for employees in the highest Purpose quintile compared to the middle (*p* = 0.000) and 39.6% higher than those working in the bottom quintile (*p* = 0.000).

The national data show that building Trust and Purpose into organizational cultures effectively improves individual and economic outcomes. Once Trust and Purpose become foci to improve organizational performance, the measures used in this study can be applied to systematically influence the Trust by intervening to raise one or more of the OXYTOCIN factors. Study 2 analyzes a business that did this to assess its effects on performance.

## Study 2: Trust Intervention at an Online Retailer

Study 1 demonstrated the effect of Trust and Purpose on multiple measures of organizational performance by analyzing a cross-section of working adults. Study 2 examines the effect of a Trust intervention in one division of a large online retailer. The longitudinal approach of Study 2 is designed test the causal impact of Trust on business-relevant outcomes.

The leaders of a large (revenue > $1 billion) online retailer (OnRet) identified low morale and high job turnover as key performance indicators they sought to affect in a division of their company. The company is very well run and even the division for which we created an intervention performs well on most metrics (Table 5). OnRet supported the intervention by inviting our team to their headquarters and having their executives participate in the kick-off meeting with 66 colleagues from the division. Data were collected prior to the intervention to measure the OXYTOCIN factors and provide baseline values for outcomes (*N* = 59). Because the OXYTOCIN factors all face ceiling effects, intervening to raise the lowest factor is expected to have a larger impact on Trust and performance than influencing a factor that is already high. The baseline data showed that the lowest factor was Natural and a 3-month intervention, described below, was designed and executed to increase Natural. After the intervention concluded, the survey was repeated to assess the OXYTOCIN factors, Trust, Purpose, and job retention. Not all variables in Study 1 could be measured in this study because OnRet’s leadership was concerned about the time colleagues would spend responding to survey questions.

**TABLE 5:** Descriptive statistics for the field experiment before and after Trust-building intervention.

**Type**	**Variable**	**Survey 1 Mean**	**SD**	**Survey 2 Mean**	**SD**
Independent	Ovation	3.372881	0.4964809	3.442308	0.3828034
Independent	eXpectation	4.084746	1.130123	4.365385	1.323312
Independent	Yield	3.194915	0.7370547	3.423077	0.5777942
Independent	Transfer	4.144068	1.110494	4.326923	1.462479
Independent	Openness	3.271186	0.6905897	3.461538	0.5276946
Independent	Caring	4.271186	1.134574	4.043846	1.385779
Independent	Invest	4.059322	1.286819	4.192308	1.435806
Independent	Natural	3.59322	1.112137	4.153846	1.222859
Independent	Trust	62.48234	10.77594	66.1859	13.72545
Independent	Purpose	4.559322	1.040264	4.782051	1.173569
Dependent	Retention	5.372881	1.01537	5.423077	1.238485
Control	Sex	0.5932203	0.4954498	0.4615385	0.5083911

### Intervention

Trust, as measured by the OXYTOCIN factors, is a set of behaviors. The intervention sought to change one of these behaviors, Natural, the ability to be one’s authentic self at work. It takes 90 days or longer of deliberate practice to change habitual behaviors and practice of the new behavior needs to be done consistently (Fogg, 2011; Dean, 2013). An effective way to create new habits is through microlearning techniques (Jaokar, 2007). These are short, intense, practice-based messages that “nudge” learners toward new behaviors.

We created 10 microlearning videos with the help of Envisia Learning (Santa Monica, CA, United States) that one of the authors wrote and narrated (PJZ). These were animated whiteboard videos that discussed the science of authenticity and asked viewers to do one new thing immediately. We also crafted 10 email reminders focusing on being Natural at work that asked employees to rate a particular aspect of Natural on a 1–7 scale. These pulse questions were designed to reinforce the new behavior. OnRet agreed to let us send their colleagues one microlearning video for 10 consecutive work days. After that, team members of this division received an email pulse question every Monday for the next 10 weeks. After a 2-month washout period to establish the stability of the changes, a survey was again sent to colleagues to assess Natural, the other OXYTOCIN factors, Trust, Purpose, and one outcome measure, job retention (*N* = 26). Due to department restructuring, not all colleagues who received the microlearning videos and email reminders completed the post-intervention survey.

### Analytical Approach

Data was analyzed through paired *t*-tests, correlations, and ordinary linear regressions. Additional results were obtained using ordered-logistic and log-log regressions.

## Results: Trust Intervention

Table 5 shows the values for the baseline and post-intervention measures and their standard deviations.

The data show that the intervention was successful at raising Natural. Only 62.7% of respondents has a positive view of Natural before the intervention compared 80.7% after (*p* = 0.041). The average value of Natural increased 15.6% (*p* = 0.02, one-tailed *t*-test) after 5 months (3 month intervention and 2 month wash-out). The change in Natural produced an increase in those rating Trust as favorable from 81.4 to 84.6% (*p* = 0.038, one-tailed *t*-test) although the average level of Trust was not statistically significant different likely due to the small sample size (change: 5.9%; *p* = 0.075; one-tailed *t*-test). The intervention also strengthened the correlations between Natural and Trust (*r*_1_ = 0.7974, *r*_2_ = 0.8846 *p* = 0.000).

The analysis also found that the intervention increased the correlations of job retention with Natural and Trust (Natural: *r*_1_ = 0.289, *r*_2_ = 0.501; Trust: *r*_1_ = 0.450, *r*_2_ = 0.655; *p*s < 0.03). As a result, we estimated a linear regression to assess the impact of Natural and Trust on job retention. We found that pre-intervention, Natural was unrelated to job retention, while Trust had a positive and significant relationship with retention. After the intervention, both Natural and Trust were both independently associated with greater job retention (*p*s < 0.05; Table 6 and Appendix).

**TABLE 6:** Natural and Trust are more strongly related to retention after the intervention while Purpose loses its significance.

**Variables**	**Retention (January)**	**Retention (June)**	**Retention (January)**	**Retention (June)**
Natural	0.0887	0.367*		
	(0.123)	(0.164)		
Trust			0.0258	0.0433*
			(0.0141)	(0.0168)
Purpose	0.396**	0.490**	0.272	0.312
	(0.131)	(0.171)	(0.146)	(0.196)
Constant	3.247**	1.552	2.524**	1.070
	(0.564)	(0.905)	(0.700)	(0.954)
Observations	59	26	59	26
R-squared	0.212	0.454	0.249	0.485
VIF	1.29	1.11	1.68	1.54

*Standard errors in parentheses. ^∗∗^*p* < 0.01, ^∗^*p* < 0.05.*

## Discussion: Trust Intervention

Study 2 sought to determine the causal effect of changing one of the OXYTOCIN factors on Trust, Purpose, and performance. The intervention increased favorable views of Natural by 28.7%. Using the values from the National sample, only a 2% change in Trust would have been expected due to the intervention, yet we found a nearly 6% increase in Trust. This was the result of the intervention increasing the average value of seven of the eight OXYTOCIN factors, though all failed to reach statistical significance. Trust begets Trust, and the increase in Natural appeared to have primed colleagues to change other trust-building behaviors.

Sample attrition is a common in field studies and the intervention faced substantial attrition. This makes it difficult to draw conclusions with confidence other than that a 3 month intervention can change one of the OXYTOCIN factors over a 5-month period. Nevertheless, the trends seen in Trust and the other OXYTOCIN factors were in the right direction and were quantitatively meaningful based on the impact on performance measures in Study 1. Trust at OnRet fell into the middle quintile of the national survey, with retention rates similar to the fourth quintile. Extrapolating from the results from Study 1, higher levels of Trust due to the intervention would have increased job satisfaction by 2.7%, life satisfaction by 1%, productivity by 1% and reduced chronic stress by 2.8%.

The design of behavioral “nudges” is consistent with the learning literature and produced the desired effect. The approach used here should be replicated in larger samples in order to fully evaluate its use in businesses. More generally, our findings on Trust and Purpose show that they can be consistently measured and managed to improve performance.

## Conclusion

The two studies in this paper show that organizational Trust and Purpose provide substantial leverage to improve business-relevant outcomes. The evidence showed that both Trust and Purpose increase productivity and earnings by employees, reduce job turnover, improve job satisfaction, and make people happier and healthier, aligning the incentives of firms and employees. We propose that companies that ignore the human element at work, falling into the trap of treating employees like capital rather than people, will become performance laggards compared to organizations that empower their workforces with Trust and Purpose.

Business is highly competitive and finding new dimensions to improve performance is an important way to sustain profits. While the effects of culture on business performance have been established, which aspects of culture matter the most is still an open issue. The studies here have identified two aspects of culture that managers can measure and manage to improve performance.

## Data Availability Statement

The raw data supporting the conclusions of this article will be made available by the authors, without undue reservation.

## Ethics Statement

The studies involving human participants were reviewed and approved by Institutional Review Board, Claremont Graduate University. The patients/participants provided their written informed consent to participate in this study.

## Author Contributions

PZ conceived and designed the survey and field experiment and collected data from field study. Both authors collected data from survey, analyzed the data, and wrote the manuscript.

## Conflict of Interest

The authors declare that the research was conducted in the absence of any commercial or financial relationships that could be construed as a potential conflict of interest.

## Appendix

### Organizational Trust and Purpose Questionnaire

Scoring: 1 = Strongly Disagree 2 = Disagree 3 = Somewhat Disagree 4 = Somewhat Agree 5 = Agree 6 = Strongly Agree 0 = Not observable/Not Applicable

1: Overall, I believe this organization is an excellent place to work.

2: My leader treats setbacks and mistakes I make as a valuable opportunity to learn and try something new.

3: My leader does not notice and demonstrate appreciation for my progress and the effort it takes to get things done well.

4: My leader takes time to listen and understand my point of view.

5: My leader can be relied upon to do the right thing even when it’s challenging or difficult.

6: I have enough time to perform and complete all the tasks required on my job.

7: I would recommend working for this organization to a relative or friend.

8: My leader expresses confidence in me or provide the necessary tools and resources to be successful on the job.

9: My leader shares timely information and knowledge freely and openly.

10: I have opportunity to develop additional skills and experiences at work.

11: My workload interferes with my personal or family time (i.e., does not allow me adequately detach and recover).

12: I am likely to stay with this organization for the next 12 months.

13: I enjoy my job.

14: I feel I am doing something worthwhile.

15: My leader does not encourage me to openly share my thoughts, suggestions and ideas.

16: My leader helps me understand how I can use my talents to professionally grow and develop further.

17: My leader meaningfully recognizes my efforts and achievements in a timely and appropriate manner.

18: My leader encourages me to do my best.

19: My leader does not provide autonomy, flexibility and control in deciding how I can make decisions and do my work.

20: My leader knows what matters to me and how best to support me.

21: My leader collectively agrees upon clear and challenging performance goals with me.

22: My leader utilizes and capitalizes on the full range of my skills, expertise, and experiences.

23: My leader is someone who confidently shares both their strengths and vulnerabilities openly and honestly.

24: I feel my work has a positive impact on the world.

25: I appreciate the values for which my organization stands.

26: Overall, I enjoy the people I work with.

**SCORING_KEY** = { a “−” indicates reverse scoring}

“Ovation” → [−3, 17], “eXpectation” → [18, 21], “Yield” → [2, −19], Transfer → [8, 22], Openness → [−15, 9], Caring → [4, 20], Invest → [10, 16], Natural → [5, 23], Joy → [26, 13], Purpose → [25, 24, 14], Stress → [6, −11], Engagement → [12, 1, 7].
